# Influence of defatted mango kernel seed flour addition on the rheological characteristics and cookie making quality of wheat flour

**DOI:** 10.1002/fsn3.825

**Published:** 2018-10-18

**Authors:** Olugbenga O. Awolu, Lakshminarayan M. Sudha, Balaraman Manohar

**Affiliations:** ^1^ Department of Food Science and Technology Federal University of Technology Akure Nigeria; ^2^ Department of Food Engineering CSIR‐Central Food Technological Research Institute Mysuru India; ^3^ Flour Milling, Baking and Confectionery Department CSIR‐Central Food Technological Research Institute Mysuru India

**Keywords:** cookies, defatted mango kernel flour, farinograph, pasting characteristics, rheology

## Abstract

Defatted mango kernel seed flour was added to wheat flour for the production of cookies. The protein contents of the wheat‐mango kernel flour blend were optimized using optimal mixture design of response surface methodology. The farinograph analysis had acceptable mixing profile at 25%‐defatted mango kernel flour incorporation, after which the mixing profile was adversely affected. The pasting temperature was between 65.8 and 75.6°C; peak viscosity ranged between 448 and 521 BU while the final viscosity ranged between 594 and 709 BU. The flour blends had promising dynamic rheological characteristics; at 15% incorporation of defatted mango kernel flour, G’ was greater than G’’ while a stage was reached when G’ was equal to G’’ at 25% incorporation. At 50% incorporation, G’’ was greater than G’. The cookies produced showed high (>70%) quality in terms of physical properties, color, and sensory evaluation at 25% incorporation of defatted mango flour.

## 1 INTRODUCTION

Baked products (bread, cookies, snacks, and cakes) continue to attract consumers’ attention worldwide due to their ready‐to‐eat nature, nutritional quality, availability, and cost‐effectiveness. Traditionally, baked products are produced from wheat. With the awakening consciousness of the need to consume health‐promoting foods by consumers worldwide, consumption of junk foods is being discouraged while researches are being focused on the development and production of functional baked products that are health‐promoting (Akubor, Onuh, & Orishagbemi, 2017; Awolu, Olarewaju, & Akinade, 2017; Awolu, Olurunfemi, Enujiugha, & Arowosafe, 2017; Bamigbola, Awolu, & Oluwalana, 2016; Sudha, Vetrimani, & Leelavathi, 2007).

Production of functional baked products involved incorporation of nutritionally rich food materials with wheat for the production of composite flours. Such food materials are rich in fiber, legumes, and phytochemicals and improved the fiber, protein, minerals, vitamins, and phytochemicals contents of the composite flour (Akubor et al., 2017; Awolu, Omoba, Olawoye, & Dairo, 2017; Bamigbola et al., 2016; Lunn & Buttriss, 2007; Singh, Rana, Sahi, Lohani, & Chand, 2012; Sudha et al., 2007). Some of the materials incorporated to enhance the nutritional qualities of baked products include cereals brans, legumes, underutilized crops (like bambara groundnut, kersting's groundnut), fruits pomace, plantain flour, moringa seed flour, and locust bean pulp (Akubor et al., 2017; Awolu, Olurunfemi, et al. 2017; Awolu, Olarewaju, & Akinade, 2017; Bamigbola et al., 2016; Sudha et al., 2007).

Cookies have been described as the most popular bakery product consumed by nearly every strata of the society (Sudha et al., 2007), and the consumption of cookies will continue to increase worldwide. Composite flours of wheat and other food raw materials rich in antioxidants, protein, and fiber are being developed in order to enhance the nutritional, physicochemical, and rheological properties (Akubor et al., 2017; Awolu, Olurunfemi, et al. 2017; Moreira, Chenlo, Torres, & Rama, 2014; Sudha, Chetana, & Reddy, 2014; Sudha et al., 2007). In fact, the development of baked products without wheat flour (also known as gluten‐free products) is on the rise. Gluten‐free‐baked products made from cereals, legumes, pomace, and fiber have led to the development of nutritionally rich and rheologically acceptable dough and baked products (Awolu, 2017; Demirkesen, Mert, Sumnu, & Sahin, 2010; Mancebo, San Miguel, Martínez, & Gómez, 2015; Sudha et al., 2016).

The increased researches into gluten‐free food materials are yet to translate into production of gluten‐free bread that is widely acceptable. This is as a result of challenges in developing gluten‐free dough with appropriate viscoelastic and rheological properties suitable for bread production. For dough to be useful for bread production, it needs to pass the tests of descriptive empirical rheological techniques, which include amylograph and extensograph (Awolu, 2018; Dobraszczyk & Morgenstern, 2003; Weipert, 1990). Farinograph provides information that is vital to baking processes such as water absorption, mixing time and intensity, stability, and consistency (Weipert, 1990), while amylograph measures the apparent viscosity and gelatinization temperature (Dobraszczyk & Morgenstern, 2003).

The elastic and viscous components of complex viscosity are separately assessed using dynamic rheology (Weipert, 1990). Dynamic rheology is measured using viscometer and rheometers; both are versatile and efficient instruments which have opened new possibilities for studying viscosity‐related problems in the science and processing of cereals (Gerth, 1980). Viscometer measures steady state flow while rheometer measures dynamic characteristics of the strain and temperature‐dependent dough. Bread dough displayed viscoelastic properties with nonlinear shear thinning and thixotropic behaviors, and their flow properties are determined using a viscometer and a rheometer (Weipert, 1990). Shear thinning is most obvious in the viscosity curves of dough. At low strains, viscosity is high and dough structure seems to be intact. In contrast, high strains lead to a large disorientation and destruction of the dough's structure and, hence, led to reduced viscosity. Dynamic oscillatory rheometers, employed a sinusoidal oscillating deformation of known magnitude and frequency to calculate the phase lag angle between stress and strain, and elastic (storage modulus, G’) and viscous (loss modulus or G’’) components of a complex viscosity η* (Weipert, 1990).

Large amounts of food wastes are produced by the food industries (about 35%), and they have been identified as serious causes of environmental problems and economic losses if not utilized effectively (Jahurul et al., 2015). These food industry by‐products can be good sources of potentially valuable bioactive compounds. Mango by‐products, especially seeds and peels, are considered to be cheap sources of valuable food and nutraceutical ingredients. Mango kernel seeds are agro‐industrial wastes with more than one million tons of mango seeds being treated as wastes annually (Karunanithi, Bogeshwaran, Tripuraneni, & Krishna Reddy, 2015). Mango kernel seeds have been shown to contain higher antioxidant activities and phenolic contents than the edible portions (Soong & Barlow, 2004). In fact, mango seed had over 70% of the total antioxidant and phenolic contents of mango fruits. Mango kernel seed flour is obtained by defatting mango seed kernels. Undefatted mango kernel seed had been shown to be rich in protein (7.53 g/100 g), fat (fiber (2.20 g/100 g), carbohydrate (69.77 g/100 g), and energy (421 KCal/100 g) and contained appreciable minerals contents (170 mg/100 g calcium, 210 mg/100 g magnesium, and 365 mg/100 g potassium) (Yatnatti, Vijayalakshmi, & Chandru, 2014).

Mango (*Mangifera indica* L.) is one of the most important fruits marketed in the world with global production exceeding 26 million tons in 2004 (FAOSTAT, 2006). It is grown naturally or cultivated mainly in tropical and subtropical regions and has been shown to be the second largest tropical fruit crop in the world (Joseph & Abolaji, 1997) behind only bananas (Jahurul et al., 2015). It is a rich source of antioxidants including ascorbic acid, carotenoids, and phenolic (Ribeiro, Barbosa, Queiroz, Knödler, & Schieber, 2008). Mango (*Mangifera indica* L.) polyphenols exhibit anti‐inflammatory and cancer‐cytotoxic properties in multiple cancer types, including malignancies of the colon and breast (Banerjee, Kim, Krenek, Talcott, & Mertens‐Talcott, 2015; Masibo & He, 2008).

Research focus has been mainly on the utilization of the mango kernel seed oil and the undefatted mango kernel seed flour. The bioactive compounds in the defatted mango kernel seed flour and the protein are left unutilized. The extract yield of phytochemical from mango kernels ranged from 1.0% (hexane extraction) to 5% (ethanol extraction) (Devi & Arumughan, 2007). The implication of this is that the amount of phytochemicals in defatted mango kernel seed flour ranged from 95 to 99%. This defatted mango kernel flour has not been utilized for product development; hence, this research is focused on the utilization of defatted mango kernel seed flour blended with wheat flour in the production of cookies. The influence of the defatted mango kernel flour on the protein content and rheological characteristics of the blends was optimized and evaluated. Cookies were developed from the blends, and the effect of the incorporation of mango kernel seed flour on the cookies quality was evaluated.

## 2 MATERIALS AND METHODS

### 2.1 Materials

Mango kernel was obtained from Mysuru, India; commercial whole wheat flour (9.1% moisture content, 11.0% protein, and 13.9% dietary fiber) by Avent Agro Pvt. Ltd, Narela, Delhi 110040, India, was sourced from Mysuru, India. Shortening (Marvo, Hindustan Lever Ltd, India), sugar powder, common salt, vanilla essence (Bush Boake), and corn flour were procured from local markets.

### 2.2 Experimental design

The experimental design for the optimization of the protein content was carried out using optimal mixture design (response surface methodology, design expert, DX 10). The independent variables were wheat flour (0–100 g/100 g) and mango kernel flour (0–100 g/100 g), while the dependent variable was the protein content. The number of replicates is shown in Table 2.

### 2.3 Determination of protein content

Protein content of the composite flour was determined using Flash 2000 N/Protein Analyzer (Thermo Scientific). About 3.5 mg sample was analyzed for the determination of the nitrogen content. The protein content was automatically evaluated by assuming a conversion factor of 6.25.

### 2.4 Rheological characteristics of wheat flour (WWF)‐defatted mango kernel flour (DMKF) blends

Blends of wheat flour and defatted mango kernel flour were prepared in the ratios 100:0 (W), 85:15 (X), 75:25 (Y), and 50:50 (Z) by replacing WWF with DMKF.

#### 2.4.1 Farinograph characteristics

The farinograph evaluation was carried out according to the standard AACC (2000) methods. The amount of water added was generated by inputting the moisture content of the composite flour into the farinograph (Model: E‐380, Brabender OHG, Duisburg, Germany)system and allowed to run for about 20 min. Farinograph testing was carried with the use of a Brabender farinograph. The flour sample of around 48 g on a 14% moisture basis was weighed and placed into the corresponding farinograph mixing bowl. Distilled water (calculated by the instrument using the water content of the sample) was added from a buret until dough was formed. As the dough is mixed, the farinograph records a curve on the graph paper depicting the farinograph parameters (Awolu, 2018). Parameters measured include water absorption, dough development time, dough stability, and mixing tolerance index.

#### 2.4.2 Pasting characteristics

The pasting characteristics were determined by using Micro‐Visco‐Amylograph (model 803201 by Brabender, Germany) according to the standard AACC (2000) methods. About 15 g of the sample was mixed with 100 mL of water. The sample was placed inside the amylograph and allowed to run for about 27 min.

#### 2.4.3 Dynamic rheology

The dynamic rheology for wheat flour‐defatted mango kernel flour blends was carried out by using modified method of Demirkesen et al. (2010) using Modular Compact Rheometer (NCR52, Anton Paar). The position was set at 2.000 mm, temperature at 25.00°C while the measuring system was PP76. The dough samples were placed between the parallel plates and the edges were carefully trimmed with a spatula. The flow experiments were carried out under steady shear condition (Shear rate ranged from 1 to 50 1/s) while for the frequency sweep test, the strain rate was kept constant below 0.5%; the dough elastic modulus and complex viscosity were measured as a function of frequency (between 0.1 and 100 rad/s).

### 2.5 Cookies preparation

The method of Sudha et al. (2007) was used to prepare cookies from the wheat flour‐defatted mango kernel flour by slightly modifying the method. The composite flour composition for cookies production and the baking conditions is presented in Table 1. After baking, cookies were cooled to room temperature, packed in polypropylene pouches, and sealed till further evaluation.

**Table 1:** Composite flour composition for cookie production and the baking conditions

Composition	Wheat: Mango kernel flour blend ratio			
	100:0 (W)	85:15 (X)	75:25 (Y)	50:50 (Z)
Flour (g)	90	90	90	90
Corn flour (g)	10	10	10	10
Salt (g)	0.5	0.5	0.5	0.5
Vanilla essence (ml)	0.5	0.7	0.8	1.0
Shortening (g)	50	50	50	50
Powder sugar (g)	40	40	40	40
Water (ml)	15	14	13	11
Baking period (min)	18	20	24	28
Baking temp (°C)	200	200	200	200

### 2.6 Evaluation of Cookies

#### 2.6.1 Physical characteristics

Method of Sudha et al. (2014) was used for the evaluation of spread (W), and thicknesses (T) of cookies were measured by placing them edge to edge and stacking, respectively. Cookies were rearranged, and measurements were made. The spread ratio W/T was calculated. The weight of the cookies was determined while the objective evaluation of texture expressed as breaking strength (g, force) was measured using the triple beam snap (three‐point break) technique of Soma, Mahadevamma, & Sudha (2016) using a texture analyzer (TAHDi, Stable Micro Systems, Godalming, UK).

#### 2.6.2 Color measurements

The color (L, a, b, and dE) of the cookies was measured using Hunter Lab's Color Flex EZ spectrophotometer (Color Flex EZ 45/O).

#### 2.6.3 Sensory evaluation

The sensory evaluation was carried out according to Sudha et al. (2014). Cookies were evaluated for color and appearance, texture, taste, and overall quality on a 9‐point hedonic scale. The scores assigned in the scorecard for these parameters were as follows: excellent—9, very good—8, good—6, satisfactory—5, fair—3, poor—2, and very poor—1.

### 2.7 Statistical analysis

The analysis of variance (ANOVA), the multifactor analysis of variance, and the multiple range test were done using the Statgraphic Centurion XVI, version 16.1.11 (StatPointInc). All analyses were carried out in triplicate. The results were expressed as mean ± standard deviation. Duncan's test at significant level of p ≤ 0.05 was applied to the data to establish the significance of the differences between the samples.

## 3 RESULTS AND DISCUSSION

### 3.1 Protein content of wheat‐mango kernel flour blends

**Figure 1:**
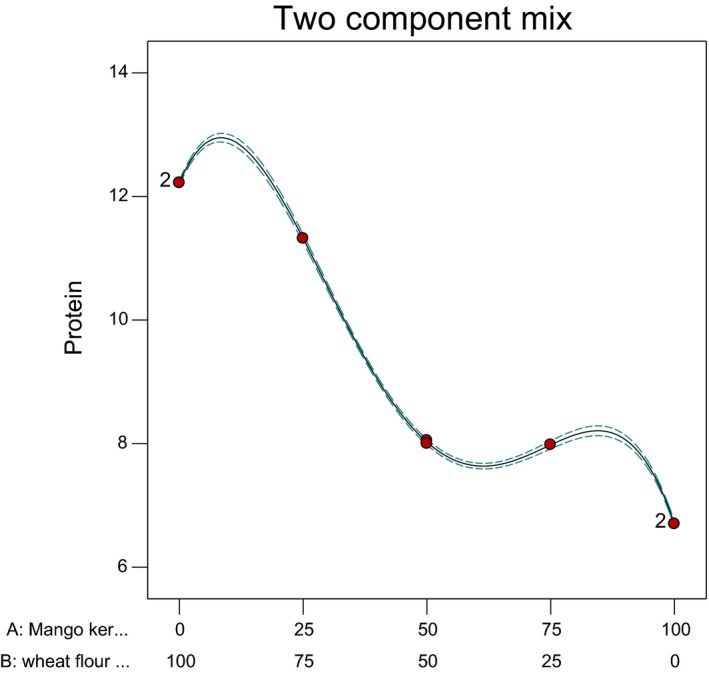
Two components mixture plot of wheat‐defatted mango kernel flour blends NB: X1 = A = Mangokernel flour X2 = B = Wheat flour

The actual and predicted protein contents of wheat‐mango kernel flour blends are presented in Table 2 while the model graph is shown in Figure 1. The results indicated that wheat flour had higher protein content than mango kernel flour. From the graph, the highest protein content could be traced to the point where the mango kernel flour was 10% and wheat flour was 90%. This implies that the addition of mango flour at 10% improves the protein content. Overall, the statistical analyses using RSM showed that the model (quartic) was significant (p ≤ 0.0001). In addition, the model terms (linear mixture, AB, AB (A−B)^2^) were significant (p ≤ 0.0001) while the model term (AB (A−B)) was significant (p = 0.0003). The *R* ^2^ and adjusted *R* ^2^ values were 1.000 and 0.9999, respectively. The implication of these results is that the composite flour consisting wheat and mango kernel flours were good sources of protein. The final equation showing the effect of the independent variables on the protein content is given in Equation (1).

**Table 2:** Protein contents of wheat‐defatted mango kernel flour blends

Run	Independent variables (g/100 g)		Dependent variable ((g/100 g)	
	Sample A	Sample B	Protein (actual)	Protein (predicted)
1	75	25	7.98	7.98
2	0	100	12.22	12.22
3	0	100	12.22	12.22
4	100	0	6.70	6.70
5	50	50	8.05	8.03
6	25	75	11.32	11.32
7	50	50	8.00	8.03
8	100	0	6.70	6.70

*Notes*

Sample A—Mango kernel flour; Sample B—wheat flour.

(1) $$\mathrm{Protein}=+6.70A+12.22B-5.74\mathrm{AB}-3.09\mathrm{AB}\left(A-B\right)+27.01\mathrm{AB}{\left(A-B\right)}^{2}$$

From the equation, the mixture of wheat and mango kernel flours at the quartic level (AB (A−B)^2^) had the highest positive coefficient, followed by sample B (wheat flour) and sample a (mango kernel flour), respectively. The highest coefficient of the mixture is a proof that wheat protein content was actually increased by the inclusion of mango kernel flour.

### 3.2 Rheological characteristics of wheat flour‐defatted mango kernel flour blends

#### 3.2.1 Farinograph characteristics

**Figure 2:**
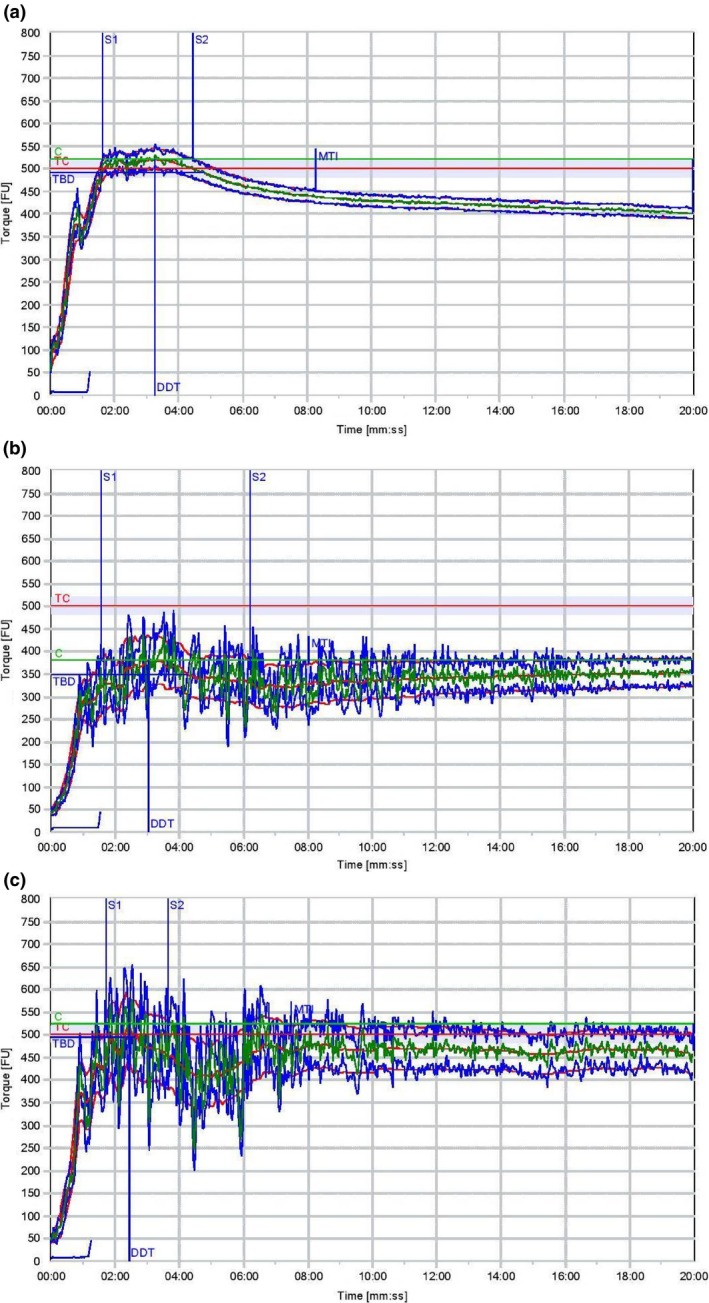
(a) Farinograph for sample W (100% wheat flour). (b) Farinograph for sample X (85% wheat flour, 15% mango kernel flour). (c) Farinograph for sample Y (75% wheat flour, 25% mango kernel flour). NB: Time is in min:s

The results of the farinograph which describe the mixing properties of the flour blends as shown in Figure 2a–c are presented in Table 3. Sample W being whole wheat had higher water absorption (76%) which is higher than the refined wheat flour (58%) reported by Sivaramakrishnan, Senge, and Chattopadhyay (2004). The water absorption of all the samples is close to refined wheat flour; the inclusion of bran in the whole wheat flour could be responsible for the value higher than 58% obtained for whole wheat flour. Inclusion of defatted mango kernel flour up to 25% had water absorption of between 59.0 and 64.7%. So, whole wheat flour supplemented with defatted whole wheat flour up to 25% has acceptable water absorption since refined wheat flour had 58% water absorption.

**Table 3:** Farinograph and pasting characteristics of wheat flour‐defatted mango kernel flour

Parameters	Sample W	Sample X	Sample Y	Sample Z
Water absorption (%)	76.0	64.7	59.0	–
Dough development time (min)	3.16	3.03	2.27	–
Dough stability (min)	2.48	4.40	1.56	–
Mixing tolerance index (FU)	93.0	62.0	53.0	–
Farinograph quality number	47.0	43.0	26.0	–
Gelatinization temperature (°C)	65.8	75.5	73.4	75.6
Peak/maximum viscosity (BU)	521.0	453.0	470.0	448.0
Cold paste viscosity (BU)	746.0	639.0	627.0	618.0
Breakdown (BU)	167.0	155.0	202.0	190.0
Setback (BU)	387.0	336.0	352.0	315.0

*Notes*

W—100% wheat flour; X—85% wheat flour + 15% mango kernel flour; Y—75% wheat flour + 25% mango kernel flour; Z—50% wheat flour + 50% mango kernel flour.

A dough development time (DDT) of 2 min was also reported for refined wheat flour (Sivaramakrishnan et al., 2004) as against values between 2.27 and 3.16 obtained for samples Y, X, and Z in this study. The results of the DDT were between 2.72 and 3.16 min. This result also indicated that wheat‐defatted mango kernel flour consisting up to 25% defatted mango kernel flour had acceptable DDT.

The farinograph curve width has been described as a measure of dough cohesiveness and elasticity. The whole wheat flour (W) had maximum cohesiveness and elasticity at the peak consistency, which reduced with increasing mixing time. Refined wheat flour also had farinogram similar to the whole wheat used in this study (Sivaramakrishnan et al., 2004). On the other hand, the farinogram width of sample Y improved in cohesiveness and elasticity with increasing mixing time. Sample Z had a very poor farinograph output; hence, the result was not reported. Results shown in Figure 2a–c showed that whole wheat flour had acceptable farinograph quality which indicated its suitability for bread production. On the other hand, the farinogram of sample X did not get to the 500 BU level which indicated it would be appropriate to be used for bread. It could, however, be suitable for other baked products production with the exception of bread. Although the farinogram of sample Y reached the 500 BU line, it, however, differs from that of sample W. Whereas sample W had acceptable farinogram which would translate to good dough quality meant for bread production, the same cannot be said of sample Y. Sample Y also with defatted mango kernel flour inclusion of 25% had better farinograph quality which, however, had lesser farinogram quality.

#### 3.2.2 Pasting characteristics

**Figure 3:**
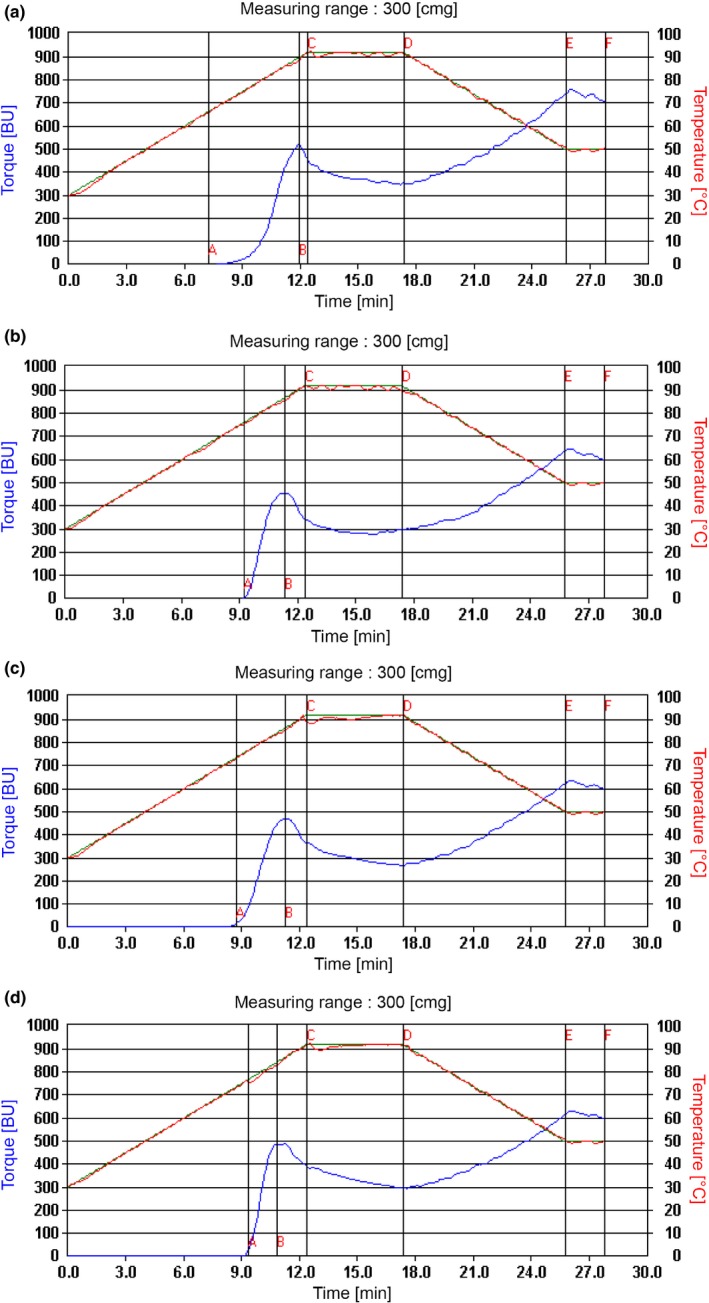
(a) Pasting profile of sample W (100% wheat flour). (b) Pasting profile of sample X (85% wheat flour, 15% mango kernel flour). (c) Pasting profile of sample Y (75% wheat flour, 25% mango kernel flour). (d) Pasting profile of sample Z (50% wheat flour, 50% mango kernel flour)

The results of the pasting characteristics of the composite flour are presented in Table 3; however, the pasting profile is shown in Figure 3. The research was able to successfully compare the pasting characteristics of the wheat‐mango kernel flour blends with that of the 100% wheat flour. The pasting temperature represents the temperature at which the viscosities first increase by at least two RVU over 20 s periods (Adegunwa, Bakare, Alamu, & Abiodun, 2012). It is an indication of the temperature required to cook the starch (Adegunwa et al., 2012), or the minimum temperature at which starch granules in the flour swell (Awolu, 2017). Sample W (100% wheat flour) had the lowest pasting temperature followed by sample (Y). Samples X, Y, and Z had close pasting temperature, probably due to the presence of mango kernel flour. The values obtained for all the samples were, however, lower than 82.2°C obtained for jackfruit seed flour (Ocloo, Bansa, Boatin, Adom, & Agbemavor, 2010) and composite flours comprising pearl millet, kidney beans, and tigernut (Awolu, 2017).

Sample W had the highest peak viscosity (521 FU) followed by sample Y, X, and Z in that order. The peak viscosity represents the pastes strength from gelatinization; it is the maximum viscosity attained during cooking (Adebowale, Sanni, & Fadahunsi, 2011). A peak viscosity value of 462 RVU obtained by Awolu (2017) for pearl millet, kidney beans, and tigernut composite flours (85:10:5) was higher than sample Z. While the 462 RVU pasting value was without wheat flour, sample Z had 75% wheat flour incorporation. In essence, the incorporation of wheat flour actually increased the peak viscosities of samples X, Y and Z.

The holding strength is the minimum viscosity after the peak, and a measure of the ability of granules to remain undisrupted during holding at high temperature (92°C) and high mechanical shear stress (Adegunwa et al., 2012). Holding period has been reported to be accompanied by breakdown in viscosity (Adegunwa et al., 2012), also known as the trough, hot paste viscosity, shear thinning, or paste stability. A lower breakdown viscosity is required, as it signifies a higher stability. Sample X had the lowest breakdown followed closely by sample W. Addition of mango kernel flour beyond 15% increased the instability of the hot pastes as revealed by the higher breakdown values.

An increase in viscosity as the temperature decreased from 92 °C to 50 °C is a reflection of the ability of the elements in pastes to associate as the paste temperature drops (Ocloo et al., 2010). This increase in viscosity is the setback viscosity. Setback viscosity corresponds to retrogradation (a realignment of the crystalline structure of starch during cooling) and leads to syneresis and staling. Unfortunately, 100% wheat flour (sample W) had the highest setback viscosity. Inclusion of defatted mango kernel flour decreased the setback viscosity. The results suggested that the incorporation of mango kernel flour had positive effects on the pasting characteristics of the wheat flour blend; the incorporation of mango kernel flour, however, reduced retrogradation by decreasing the setback viscosity.

#### 3.2.3 Dynamic rheology

**Figure 4:**
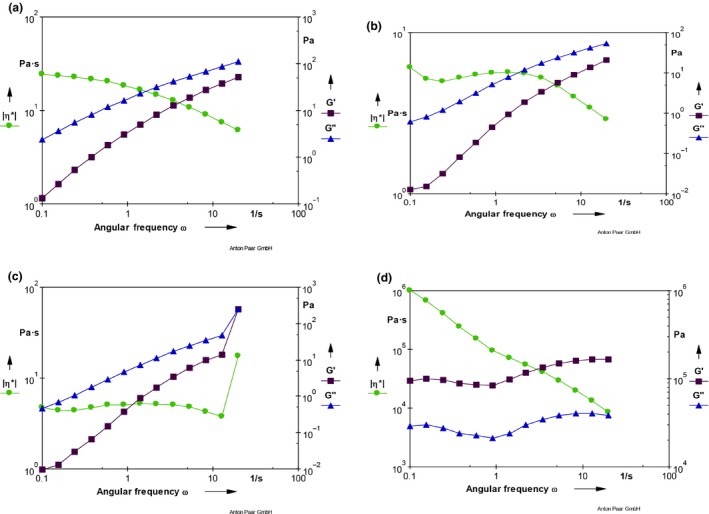
(a) Storage modulus (G’), loss modulus (G’’), and complex viscosity for sample W (100% wheat flour). (b) Storage modulus (G’), loss modulus (G’’), and complex viscosity for sample Y (75% wheat flour, 25% mango kernel flour). (c) Storage modulus (G’), loss modulus (G’’), and complex viscosity for sample Z (50% wheat flour, 50% mango kernel flour). (d) Storage modulus (G’), loss modulus (G’’), and complex viscosity for 100% mango kernel. NB: η* = Complex viscosity G′ = Storage modulus G′′ = Loss modulus

The results of storage modulus (G’), loss modulus (G’’) and complex viscosity are shown in Figure 4a–d. There is similarity in Figures 4a (100% wheat flour) and 3b (75% wheat flour, 25%). In both cases, loss modulus (G’’) is greater than storage modulus (G’) which implies they tend to be more viscous than elastic. Similar results reported showed that high water content in a dough resulted in G’’ greater than G’ (Autio, Flander, Kinnunen, & Heinonen, 2001; Mancebo et al., 2015). Also, a higher G’’ was reported in gluten‐free dough (Ronda, Pérez‐Quirce, Angioloni, & Collar, 2013). Higher G’’ has been reported to indicate formation of weak gel‐like structure. It has been established that water added to doughs mainly has strong plasticizing effects, but does not modify the supramolecular organization of high molecular weight proteins (Berland & Launay, 1995). Berland and Launay (1995) also reported that increase in water content only leads to softening of the dough (G’, G”, and η* decrease) as observed in this work but does not alter dough structure. Bread dough has been shown to displayed viscoelastic properties with nonlinear shear‐thinning and thixotropic behaviors (Weipert, 1990). So, the shear‐thinning behavior obtained in this study could be said to be appropriate for bread production dough. In addition, low strains had been shown to produce high viscosity (dough structure seems to be intact), and vice versa. This study also had similar behavior with high viscosity at low strain (Figure 4).

**Figure 5:**
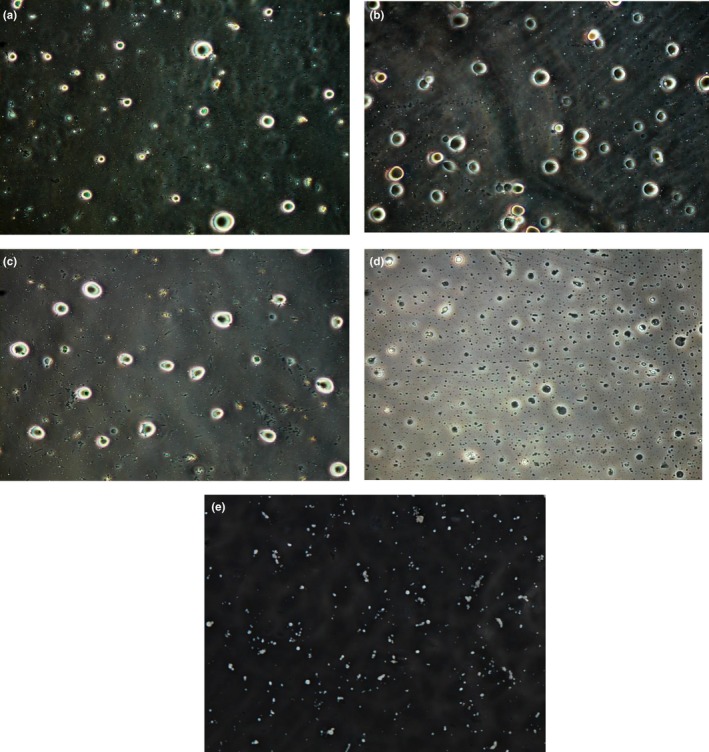
Microscopic images (×100) of (a) acetone extracted mango kernel oil, (b) ethanol extracted mango kernel oil, (c) hexane extracted mango kernel oil, (d) Petroleum ether extracted mango kernel oil, (e) SC‐CO2 extracted mango kernel oil

Increase in the incorporation of mango seed flour (50%), however, reversed the dynamic rheology trend observed in 100% wheat flour. An equal G’ and G’’ were obtained as the angular frequency increased in sample Z (Figure 4c), while pure mango kernel flour, the G’ was greater than G’’. This indicates that mango seed incorporation would promote elasticity of wheat flour, especially when the water content of the dough is high. The complex viscosity has been reported to decrease with the frequency (Liu, Wang, Liu, Zhou, & Luo, 2015). The shear stress versus shear strain plot produced pseudoplastic (shear‐thinning) curves (Figure 5a–c).

### 3.3 Characterization of Cookies

The results of the physical characteristics of the cookies are presented in Table 4. There was no significant difference (p ≤ 0.05) in cookie diameter of samples W, Y, and Z; there was, however, a significant difference (p ≤ 0.05) in cookie diameter of sample X and others. There were significant (p ≤ 0.05) differences in the thickness of the cookies. Wheat flour had the highest thickness; the incorporation of DMKF significantly (p ≤ 0.05) reduced the cookies thickness. The same trend was also observed in the weight of the cookies. However, cookies made from wheat‐mango kernel flour blends had higher breaking strength. The breaking strength of cookies increased by twofold as the incorporation of DMKF increased from 15 to 50%. Breaking strength of cookies at 25% DMKF was close to wheat flour cookies. The results of the color (Lab) measurement of the cookies are presented in Table 4. Positive L* represents lightness, positive a* represents redness, and positive b* represents yellow. The results indicated acceptable cookies color. Expected color for the cookies is golden brown for the surface and creamish/yellowish white for the crumb. Inclusion of defatted mango kernel flour up to 25% in the cookies had color properties reasonably comparable to whole wheat flour cookies.

**Table 4:** Physical characteristics of wheat flour‐defatted mango kernel cookies

Parameters	Sample W	Sample X	Sample Y	Sample Z
Physical characteristics				
Diameter (cm)	7.00^a^	6.94^b^	7.04^a^	7.03^a^
Thickness (cm)	1.28^a^	1.20^c^	1.18^d^	1.25^b^
Weight (g)	32.48^a^	32.20^b^	30.45^d^	31.83^c^
Breaking force (kg)	3.26^c^	4.61^b^	4.88^b^	7.40^a^
Color				
L*	57.64^b^	59.58^a^	57.55^b^	54.45^c^
a*	4.40^b^	3.55^c^	4.70^ab^	15.60^b^
b*	15.97^a^	15.94^a^	16.01^a^	15.60^b^
dE	38.04^b^	36.14^c^	38.18^b^	40.99^a^
Sensory evaluation				
Surface color (9)	8.00^a^	8.10^a^	7.10^b^	5.50^c^
Surface character (9)	7.55^a^	7.55^a^	7.15^ab^	6.50^b^
Crumb color (9)	7.55^a^	7.05^b^	7.10^b^	5.75^c^
Texture (9)	8.05^a^	7.55^ab^	6.65^b^	5.50^c^
Mouthfeel (9)	8.10^a^	7.60^ab^	6.60^b^	5.50^c^
Overall quality (9)	8.05^a^	7.60^ab^	7.10^b^	5.00^c^

*Notes*

Values in the parenthesis for sensory evaluation indicate maximum score; Values for a particular row followed by different letters as superscript differ significantly (p < 0.05). W—100% wheat flour; X—85% wheat flour + 15% mango kernel flour; Y—75% wheat flour + 25% mango kernel flour; Z—50% wheat flour + 50% mango kernel flour.

The sensory evaluation (Table 4) showed that there were significant (p ≤ 0.05) differences in the cookies from samples W, X, and Y in terms of surface color, crumb color, texture, mouthfeel, and overall quality assessment. Samples X (15% mango kernel flour) and Y (25% mango kernel flour) incorporation had sensory evaluations similar to 100% wheat flour (sample W). It could therefore be inferred that incorporation of up to 25% mango kernel flour into wheat flour resulted in cookies with acceptable sensory properties. Wheat‐mango kernel flour blend of about 50:50 resulted in unacceptable sensory evaluations.

## 4 CONCLUSIONS

Incorporation of defatted mango kernel flour into wheat flour would be suitable for production of baked products. The protein content was nutritionally sufficient while the rheological evaluation showed that wheat‐defatted mango flour blends up to 25% defatted mango flour addition were adequate; they displayed acceptable dough mixing and viscoelastic behavior. In fact, addition of defatted mango flour enhanced the elastic characteristic of the flour blends. The pasting properties of the blends were similar to 100% wheat flour, indicating a good starch heating properties. The cookies at 25% incorporation of defatted mango kernel flour showed an excellent (>70%) physical and sensory properties as well as acceptable measurement. The overall cookie quality acceptability at 25% defatted mango kernel flour incorporation was over 70%.

## CONFLICT OF INTEREST

Authors declare there is no conflict of interests.

## ETHICAL REVIEW

This study does not involve any human or animal testing.
